# The passage of time during the UK Covid-19 lockdown

**DOI:** 10.1371/journal.pone.0235871

**Published:** 2020-07-06

**Authors:** Ruth S. Ogden

**Affiliations:** School of Psychology, Liverpool John Moores University, Liverpool, England, United Kingdom; UCLA Fielding School of Public Health, UNITED STATES

## Abstract

In March 2020, in response to the Covid-19 pandemic, the UK Government imposed social and physical distancing measures on the population. These lockdown measures caused significant changes to all aspects of daily life. The current study examined how the passage of time was distorted during the lockdown period. Using an online questionnaire, day and week passage of time judgments were collected. In addition, measures of affect, task load and satisfaction with current levels of social interaction were taken. The results show that over 80% of participants experienced distortion to the passage of time during lockdown in comparison with normal. The passage of time during the day was predicted by age, stress, task load and satisfaction with current levels of social interaction. A slowing of the passage of time was associated with increasing age, increasing stress, reduced task load and reduced satisfaction with current levels of social interaction. Only age and satisfaction with current levels of social interaction predicted passage of time across a week. Again, increasing age and reduced satisfaction with levels of social interaction were associated with a slowing of the passage of time. These findings demonstrate that significant changes to daily life have a significant impact on our experience of time, with younger, more socially satisfied people more likely to experience time as passing more quickly during the lockdown.

## Introduction

The 2020 novel Coronavirus-19 (Covid-19) pandemic, and the subsequent attempts to contain the virus, have resulted in significant changes to daily life in the United Kingdom. On the 23^rd^ of March 2020, to reduce the transmission of Covid-19, the UK Government imposed measures which placed significant restrictions on the circumstances in which members of the public could leave their homes. Members of the public were instructed that they should only leave the house for very limited purposes, including (1) shopping for basic necessities, for example food and medicine, which must be as infrequent as possible, (2) one form of exercise a day, for example a run, walk, or cycle—alone or with members of their household, (3) any medical need, including to donate blood, avoid or escape risk of injury or harm, or to provide care or to help a vulnerable person, and (4) travelling for work purposes, but only where they cannot work from home. Failure to comply with these rules could result in a fine or arrest. When people did leave the house, they must stay at least two meters away from people who do not live in their household. These social and physical distancing measures have been termed “the lockdown” by the British public and media.

The societal and behavioural changes as a result of these measures have been significant. Children, except those of key workers, are unable to attend school. Homeworking has increased, as has unemployment and the temporary furloughing of employees. People residing in different households are no longer permitted to interact with one another, severely limiting the opportunity for face-to-face socialization for many people. Such substantial changes to daily live will have significant consequences for the way in which people experience the world around them. This study aimed to establish how the passage of time was experienced during the Covid-19 lockdown in the UK.

### Time experience

Objectively time passes at a constant linear rate. Subjectively, however, our experience of time is influenced by the activities that we perform and the emotions that we experience [1]. To date, the overwhelming majority of studies examining human time perception have taken place in laboratories and have focused on the processing of short (< 1 minute) durations. These studies have provided clear evidence that emotion [2, 3], cognitive capacity [4, 5], psychopathology [6, 7], drug administration [8] and cognitive load [9] can systematically distort the perceived duration of short events. Relatively few studies however have explored people’s experience of time, outside of the laboratory, in the real world [10, 11].

Passage of time judgments (POTJ), describe the subjective speed at which time feels as though it is passing, for example, more quickly than normal, as normal, or more slowly than normal [1, 10–13]. POTJs can be made in the moment, for example, how quickly does time feel as though it is passing in this moment, sometimes referred to as a present judgment of passage of time [14, 15], or they can be made retrospectively over longer epochs, for example, how quickly did the previous hour, day, week or month feel like it was passing in comparison with normal [1]. Although POTJs are temporal judgments, they appear to be based on different mechanisms to those involved in the perception of short (sub-second) durations [10, 15, 16] and therefore warrant research in their own right. The social and physical distancing measures resulting from the Covid-19 lockdown present an opportunity to examine how the passage of time is affected by the significant changes to daily life described above.

The idea that significant deviation from “normal” may affect the passage of time is not new. Previous research has examined the effects of being confined to a nuclear fallout bunker [17, 18] or self-isolating alone in a cave for a period of weeks or months [19]. In these situations, life was significantly different to normal and significant distortions to the passage of time were observed. However, these studies were essentially experimental in nature, participants did not have any access to external temporal markers, for example, watches, clocks or even natural light. Consequently, the findings perhaps reflect the effect of a loss of external time references rather than solely the effects of disruption to normal life. There is however, very limited evidence to suggest that the passage of time can distort during periods of social atypicality. Flaherty Freidin, and Sautu (2005) [20] interviewed people about the passage of time during the 5 months after the 2001 resignation of President Fernando de la Rúa. Distortions to the passage of time were common. A fast passage of time was associated with the rapidly changing domestic situation following the resignation whereas slow time was associated with economic and personal insecurity and suffering. Further research which quantifies the predictive factors which contribute to distortions to the passage of time during periods of profound social change is however warranted.

Studies of the passage of time during “normal” daily life offer insights into how and why the social and physical distancing measures imposed during Covid-19 may affect how an individual experiences the passage of time. The adage “*time flies when you’re having fun*” suggests that our emotional state influences the subjective speed with which time passes. This suggestion is supported by studies showing that self-reported mood and arousal are predictive of the speed of the passage of time during real-world activity and in laboratory studies. In a series of studies, Droit-Volet and colleagues have consistently demonstrated that, for present POTJs, positive affect and higher arousal are associated with the sensation of time passing more quickly in the moment [14, 15, 21, 22]. Wearden et al., (2014) [1] also reported an association between positive affect and time passing more quickly than normal when studying people’s recollections of the passage of time during drug use, suggesting that the association is not dependant on the judgment being made in the present moment. Indeed, it has been suggested that the sensation of time flying may act as a cue for enjoyment of an experience [23].

Whilst positive affect and high arousal are associated with time passing more quickly than normal, negative affect and low arousal are associated with time passing more slowly than normal. People with depression report that time passes more slowly than normal during episodes of depression [24–26]. Time also passes more slowly than normal for hospitalized oncology patients, with lower levels of wellbeing being associated with greater slowing of time [27]. In non-clinical populations, self-reported negative affect (sadness) and low levels of arousal are predictive of a slowing of the passage of time in the present moment [14, 15, 21] and when retrospectively judging the passage of time during previous life events [1]. Boredom has also been consistently associated with a slowing of the passage of time in a range of laboratory and real-world studies [1, 28–30]. Conversely however, time is also reported to slow down in situations of mortal peril, for example, car accidents, suggesting that the relationship between arousal and the passage of time may be complex [31].

The UK lockdown restrictions are likely to have significant effects on the mental health and affective experiences of the nation [32]. Indeed, a study of mental health in China during its lockdown measures suggests that anxiety, depression and panic all increased during this time [33]. Emotional changes as a consequence of UK’s lockdown may therefore alter the subjective passage of time during this period in comparison to normal.

The subjective speed at which time passes is also influenced by the physical and cognitive load of the tasks we perform. In general, research shows that performing familiar tasks, or tasks which have a low cognitive load, results in the sensation of time passing more slowly than normal. Increases in task complexity, the intellectual requirements of the task and the level of task engrossment, are all associated with time passing more quickly [1, 10, 11, 16, 34]. However, because time can pass also more slowly when task requirements exceed resources, Flaherty (1993) [35, 36] proposed a U-shaped relationship between the complexity of an event and the speed at which time appears to pass, with extremely high and low levels of complexity leading to a slowing of the passage of time. These effects may arise from differing levels of attention to time during different levels of task load. When load is very low there is spare cognitive capacity to focus attention on the passing of time and a greater than average level of attention to time may result in the sensation of it passing more slowly, as is observed during boredom [11]. Conversely, when load is high, attentional resources are dedicated to the task and little attention is paid to time, this may contribute to the sensation of time passing more quickly than normal. Changes in daily task load, because of changing work patterns, unemployment and increased childcare requirements, seem a likely consequence of the UK lockdown and it therefore possible that these changes will distort the passage time during this period.

Finally, there is inconsistent evidence that age influences our experience of the passage of time. Increasing age is often associated with a feeling of an acceleration in the subjective speed of time [37–41]. For example, studies in which people are asked to reflect on the speed of the passage of time now in comparison with earlier periods of their life suggest that the passage of time accelerates with age [39, 41]. However, evidence from direct comparisons of the subjective speed of time in different age groups is more equivocal. When comparing the relative speed of time between groups of different ages, older groups are only consistently found to experience “the last 10 years” as passing more quickly than younger groups, with experiences for other epochs (e.g. the day or week) appearing to be similar [37, 38, 40].

Studies conducted during real world activity have however failed to demonstrate a consistent effect of age on the passage of time. Droit-Volet and Wearden (2015; 2016) [14, 15] observed no differences between present judgments of passage of time (how quickly does time feel like it is passing in the present moment) for young people and older individuals. In a further study, Droit-Volet (2019) [21] questioned whether age effects only emerge in the very elderly. After comparing the passage of time in the over 75s with a younger group of elderly people (aged 64–75 years), she observed that the present passage of time was perceived as slower in people over the age over 75 years in comparison to the younger group of elderly people. Droit-Volet (2019) [21] however failed to observe any consistent differences in the participants’ POTJ judgments for the subjective speed of time over the previous day, week, month or year. It is possible that differences in POTJ judgments may have been observed if a much younger comparison group had been included.

If the passage of time is affected by age, it is possible that time experience will differ for younger and older people during the lockdown. Indeed, because the lockdown measures may differentially impact on different age groups, the potential for age effects on POTJ to emerge may be greater in this period than under normal circumstances. For example, because old adults are at greater risk of mortality from Covid-19 [42] and are likely to live alone [43], greater levels of social isolation and stress may make temporal distortion more likely, or more extreme, for them than younger individuals.

The studies discussed above demonstrate that the subjective speed at which time passes varies during normal day-to-day life. What remains unclear however, is how the passage of time distorts when there is a significant change in the routine of day-to-day life through social and physical distancing. The current study sought to establish how the passage of time was experienced during the 2020 Covid-19 lock-down period. Specifically, the study aimed to establish whether the subjective speed at which time passed during the lockdown differed from normal. It also aimed to establish factors which influenced the subjective speed of the passage of time.

An online questionnaire was developed to capture data on reported passage of time during lockdown. The questionnaire contained two passage of time judgements questions, one exploring passage of time during that day (POTJ-day) and another exploring passage of time over the last week (POTJ-week). Both questions required participants to indicate how quickly time felt like it was passing in comparison to normal using a 7 point likert scale ranging from 1, extremely slowly to 7 extremely fast. Other measures in the questionnaire were informed by the literature reviewed above. Previous research shows a clear effect of affective experience on POTJs. Affect was therefore measured using the DASS-21 [44] which provides measures of depression, anxiety and stress. Load, which has also been demonstrated to influenced POTJ, was assessed using a modified version of the NASA-TXL [45] in which participants were asked to respond on the basis of their average day during lockdown. In addition, because the UK lockdown restrictions heavily focus on social and physical distancing, participants were asked to indicate 1) their level of satisfaction with their social interactions since lock-down, 2) their level of physical activity since lockdown and 3) the extent to which they agreed that their daily lives had changed since lockdown. Participants also provided demographic details including age, gender, employment status, number of cohabitants and perceived risk from Covid-19.

A number of hypotheses were developed, based on the research discussed above. Firstly, it was expected that, due to the significant lifestyle changes imposed by the lockdown, the passage of time would be distorted relative to normal. Secondly, it was expected that the relative distortion to the passage of time would be associated with measures of affect including depression, stress, anxiety and satisfaction with current levels of socialisation. Specifically, it was expected that greater levels of depression, stress and anxiety, and lower levels of satisfaction with social interaction, would be associated with a slower passage of time, whereas greater levels of satisfaction with socialisation and lower levels of depression, anxiety and stress would be associated with a faster passage of time. Thirdly, it was expected the task load would also be associated with the passage of time, with a greater average daily task load being associated with a faster passage of time. Finally, because Covid-19 poses a greater risk to the elderly and because the current social and physical distancing measures are likely to have a significant impact on the elderly and those living alone, it was expected that a slower passage of time would be associated with increasing age and living alone.

## Methods

### Participants

687 participants were recruited through volunteer sampling via email and social media advertising. Eighty-four were excluded from the study because they failed to answer one or more questions, or because they were not currently residing in the UK. This left a final sample of 604 participants with complete datasets. Table 1 shows demographic information. The study was approved by Liverpool John Moores University Research Ethics Committee (ref 20/NSP/01) and all participants gave informed written consent. The study was conducted in accordance with the principles expressed in the Declaration of Helsinki.

**Table 1:** Descriptive statistics of the proportion of participants in different demographic groups and the mean POTJ for each group.

	Mean (SD) %	Mean POTJ-day (SD)	Mean POTJ–week (SD)
*Age (years)*	34.88 (14.24)		
*Young < 26*	36.30	4.37 (1.82)	4.55 (1.94)
*Middle aged*	55.80	4.15 (1.61)	4.25 (1.75)
*Older >60*	7.90	3.29 (1.54)	3.33 (1.62)
*Gender*			
*Male*	25.50	4.23 (1.62)	4.28 (1.74)
*Female*	75.50	4.13 (1.73)	4.28 (1.86)
*Cohabitation status*			
*Living alone*	8.80	3.79 (1.68)	3.89 (1.92)
*Cohabiting*	91.20	4.20 (1.71)	4.32 (1.83)
*In an at risk group*			
*Yes*	16.60	4.00 (1.83)	4.11 (2.03)
*No*	70.00	4.18 (1.69)	4.31 (1.80)
*Unsure*	13.70	4.27 (1.65)	4.34 (1.78)
*Employment status*			
*Employed full time*	40.70	4.09 (1.63)	4.19 (1.79)
*Employed part time*	11.80	4.18 (1.52)	4.45 (1.71)
*Unemployed looking for work*	2.00	4.67 (1.78)	4.92 (2.11)
*Unemployed not looking for work*	2.00	4.33 (1.88)	4.33 (1.88)
*Retired*	2.80	3.18 (1.24)	3.00 (1.23)
*Student*	33.10	4.18 (1.84)	4.36 (1.8 9)
*Disabled*	.70	4.25 (1.71)	3.75 (2.06)
*Furloughed*	6.80	4.59 (1.76)	4.56 (1.99)
*Depression*	6.40 (5.11)		
*Normal*	41.40	4.43 (1.52)	4.53 (1.57)
*Moderate*	39.20	4.07 (1.73)	4.22 (1.93)
*Severe*	19.40	3.77 (1.94)	3.89 (2.09)
*Anxiety*	3.30 (3.45)		
*Normal*	63.60	4.22 (1.59)	4.29 (1.71)
*Moderate*	24.70	4.19 (1.87)	4.52 (1.99)
*Severe*	11.80	3.80 (1.92)	3.77 (2.05)
*Stress*	7.15 (4.65)		
*Normal*	58.80	4.29 (1.57)	4.38 (1.69)
*Moderate*	26.30	4.17 (1.83)	4.37 (1.97)
*Severe*	14.90	3.63 (1.90)	3.67 (2.07)
*Task-load*	17.43 (3.76)		
*Social satisfaction*	2.65 (1.17)		
*Physical activity*	3.05 (1.22)		
*Changed routine*	4.49 (.81)		

### Measures

Participants completed an online questionnaire distributed through Qualtrics.com. The questionnaire was released to participants on the 7^th^ of April 2020, 14 days after the commencement of the lockdown and closed on the 30^th^ of April 2020. Participants had therefore experienced between 14 and 38 days of lockdown at the time of participation. The questionnaire recorded demographic information, average level of physical activity, satisfaction with socialisation, perceived risk, passage of time judgements. Mood was assessed using the DASS-21 [44] and average daily task load was assessed using a modified version of the NASA-TLX [45]. Participants took approximately 5 minutes to complete the questionnaire. See S1 File for a copy of the questionnaire.

#### Demographic questions

Participants stated their age, gender, employment status, whether they were in a high-risk category for Covid-19 and how many people they lived with.

#### Passage of time judgements

The following questions were posed about the daily and weekly passage of time.

1. *“Thinking about today*, *how quickly has time felt like it is passing in comparison with normal (i*.*e*. *before lockdown)*?*”*
2. *Thinking about this week*, *how quickly has time felt like it was passing in comparison to normal (i*.*e*. *before lockdown)*?

Participants responded using the following 7 point Likert scale: 1. Extremely slow, 2. somewhat slower, 3. a little slower, 4. as normal, 5. a little faster, 6. somewhat faster, 7 extremely fast. A higher score therefore indicated a faster passage of time.

#### DASS-21

The DASS-21 is a short version of the 42-item Depression Anxiety Stress Scales [44], which measures depression, anxiety, and stress. The 21 item questionnaire contains three, seven item subscales measuring depression, anxiety and stress. Responses are provided by indicating the severity with which each item reflected the participants experience: (1) did not apply to me at all; (2) applied to me to some degree; (3) applied to me to a considerable degree; and (4) applied to me very much. The DASS-21 is not a diagnostic tool, however, scores from the DASS-21 can be doubled to enable classification as normal, moderate or severe using the following cut-offs: depression; normal < 9, moderate 10–20 and severe > 21, anxiety; normal < 6, moderate 9–14 and severe > 15 and stress; normal < 10, moderate 11–26 and severe > 27. Cronbach’s alpha for the 21 item DASS questionnaire was 0.93.

#### National Aeronautics and Space Administration-Task Load Index (NASA-TLX)

The NASA-TLX [45] is an extremely widely used measure of subjective workload. The NASA-TLX measures subjective workload using six single item questions measuring: mental demands, physical demands, temporal demands, personal performance, effort and frustration. In the current study, a modified version of the NASA-TLX was used to assess the subjective workload of an average day during the period of lock-down. Participants were asked to rate each of the six items, in terms of their average day during the lock-down period, using a 5 point Likert scale in which a high score indicated greater task demands. Cronbach’s alpha for NASA-TXL questionnaire was 0.64.

To measure social satisfaction participants were asked to rate how “Since the Covid-19 lockdown, how satisfied are you with your daily level of social interaction?” using a 5 point Likert scale in which a high score indicated greater satisfaction. To measure physical activity, participants rated “Since the Covid-19 lockdown, how would you describe your level of physical activity? Using a 5 point Likert scale in which a high score indicated greater activity. Finally, participants also used a 5 point Likert scale to rate to what extent they agreed that: "My daily routine has changed a lot as a result of the Covid-19 lockdown? Here, a high score indicated greater agreement.

### Data analysis

Because the main dependant variables, POTJ-day and POTJ-week, were ordinal scales, non-parametric analyses were conducted. Kruksal-Wallis tests were used to establish the effect of age, gender, personal risk and cohabitation status on POTJs. In this analysis, age was classified into three groups; young adults (25 years and under), middle aged adults (26–59 years) and older adults (aged 60 years and over). These groups were based on those used in the existing literature examining the effect of age on time judgments [14, 15, 37, 46]. To assess the relationship between the passage of time and measures of affect (DASS-21 depression, anxiety and stress scores, satisfaction with social interaction), task load (NASA-TXL scores and rating of physical activity) and age, Spearman’s correlations were conducted. Finally, to assess whether these factors were predictive of POTJ’s, separate ordinal logistical regression analyses were conducted for POTJ-day and POTJ-week.

## Results

**Fig 1:**
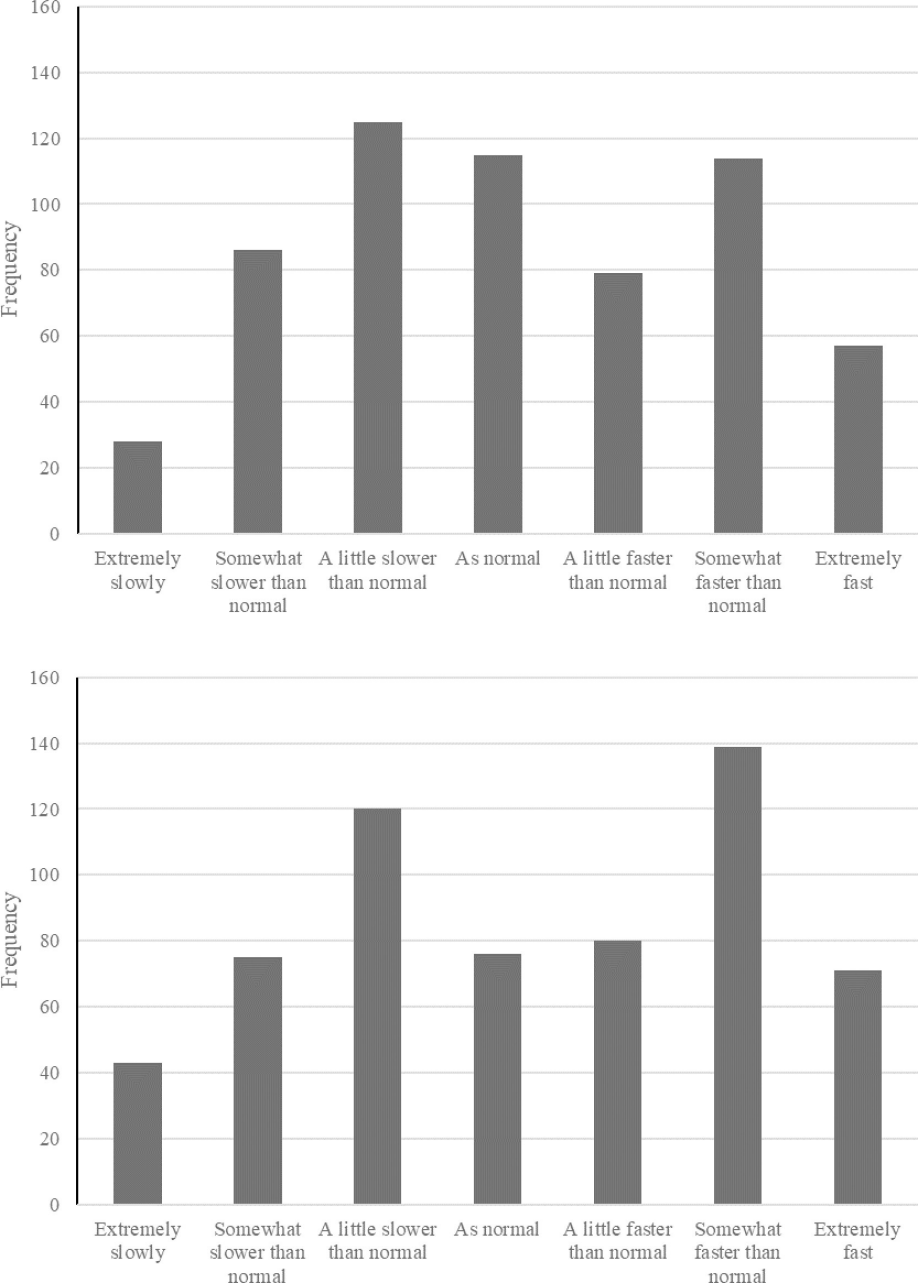
The frequency of responses for each Likert point for the day passage of time judgement (upper panel) and week passage of time judgment (lower panel).

Fig 1 shows the distribution of responses for the day (upper) and week (lower) passage of time judgments. Examination of Fig 1 suggests that distortion to time was prevalent during the period of study. For POTJ-day, only 19% of participants reported that time was passing at a normal rate, the figure was 13% for POTJ-week. Instead, for POTJ-day 40% of participants reported that time passed more slowly than normal and 41% reported than time passed more quickly than normal. For POTJ-week, 39% of participants reported that time passed more slowly than normal and 48% reported than time passed more quickly than normal. Spearman’s Rho correlation showed a significant positive relationship between POTJ-day and POTJ-week *r*(602) = .82, *p* < .001.

Table 1 shows mean passage of time judgments expressed as a function of age group, gender, personal risk, occupation and cohabitation status. Kruksal-Wallis tests were used to analyse this data. There was a significant effect of age group on POTJ-day χ^2^(2) = 14.40, *p* = .001. Post-hoc Mann Whitney-U tests confirmed a significantly slower passage of time for older adults than middle aged adults (*p =* .002) and young adults (*p <* .001). There was no significant difference in the passage of time for middle aged and young adults (*p =* .11). There was also a significant effect of age on POTJ-week χ^2^(2) = 17.63, *p* < .001. Post-hoc Mann Whitney-U tests confirmed significantly slower passage of time for older adults than middle aged adults (*p <* .001) and young adults (*p <* .001). There was no significant difference in the passage of time for middle aged and young adults (*p =* .38). Therefore, for both POTJ-day and POTJ-week, whist being over the age of 60 was associated with a slowing of the passage of time, people under the age of 60 appear to experience the passage of time comparably.

There was no effect of gender on POTJ-day χ^2^(2) = 1.85, *p* = .40 or POTJ-week χ^2^(2) = 2.41, *p* = 0.30. There was no effect of perceived risk from Covid-19 on POTJ-day χ^2^(2) = 1.13, *p* = .57 or POTJ-week χ^2^(2) = 0.85, *p* = 0.65. There was no effect of cohabitation status on POTJ-day χ^2^(2) = 5.65, *p* = .06 or POTJ-week χ^2^(2) = 3.96, *p* = 0.14. There was also no effect of current occupation on POTJ-day χ^2^(7) = 8.71, *p* = .27 or POTJ-week χ^2^(7) = 12.33, *p* = 0.09.

Table 2 shows correlation coefficients for the relationships between POTJs and measures of affect, task load and change in daily routine. There were significant negative relationships between POTJ-day, age, depression, stress. There was a positive relationship between satisfaction with social interactions and POTJ-day. Slower daily passage of time was therefore associated with greater age, greater levels of depression and stress and lower satisfaction with social interactions. There were also significant negative relationships between POTJ-week and age, depression and stress. Satisfaction with social interaction was positively related to POTJ-week. Slower weekly passage of time was therefore associated with greater age, greater levels of depression and stress and lower levels of social satisfaction.

**Table 2:** Correlation coefficients between POTJs, measures of affect, load and change to life.

	*Day POTJ*	*Week POTJ*	*Change to daily life*	*Level of physical activity*	*Task load*	*Social satisfaction*	*Stress*	*Anxiety*	*Depression*	
Age	-.10*	-.13**	-.01	.15**	.06	.22**	-.27**	-.22**		-.28**
*Depression*	-.16**	-.13**	.10*	-.34**	-.10*	- .44**	.70**	.60**		
*Anxiety*	-.04	-.01	.10*	-.23**	.09*	-.26**	.68*			
*Stress*	-.11**	-.08*	.11*	.24**	.15*	-.35*				
*Social satisfaction*	.19**	.16**	-.19**	.26**	.10					
*Task load*	.09	.07	-.07	.19**						
*Level of physical activity*	.09	.06	-.01							
*Change to daily life*	-.04	-.05								

* = *p* < .05

** = *p* < .001.

Table 2 also shows the relationship between measures of affect, load and change to normal routine. Notably, increasing age is associated with lower levels of depression, anxiety and stress but high levels of satisfaction with social interaction. Greater social dissatisfaction was associated with and reducing physical activity were also associated with greater levels of depression, anxiety and stress. Finally, greater change to daily life as a result of Covid-19 was associated with greater depression, anxiety and stress and reduced satisfaction with social interactions.

Ordinal logistic regression with proportional odds was conducted to establish the effect of demographic factors, measures of affect and task load on POTJ-day and POTJ-week separately. Table 3 shows the odds ratios for each variable with 95% confidence intervals. For the POTJ-day, the model was a statistically significant, χ^2^(19) = 68.01, *p* < .001 fit for the data, with pseudo R squared values of .03 - .11. For the POTJ-week, the model was a statistically significant, χ^2^(19) = 58.46, *p* < .001 fit for the data with pseudo R squared values of .03 - .10. Table 3 shows that, for POTJ-day, there were significant four predictor variables; the likelihood of perceiving the passage of time as slow increased with increasing age, increasing stress, decreasing task load, and greater dissatisfaction with social interactions. For POTJ-week, only age and satisfaction with social interaction were significantly predictive. Again, increasing age and reducing satisfaction with current levels of social interaction were associated with a slowing of the passage of time.

**Table 3:** Wald, odds ratios and 95% confidence intervals from the ordinal regressions for POTJ-day and POTJ-week.

		*POTJ-day*			*POTJ-week*		
		*Wald*	*Odds Ratio*	*95% CI*	*Wald*	*Odds Ratio*	*95% CI*
*Age*		13.50	.97**	.96 -.99	13.41**	.97**	.96 –.99
*Gender*	Female (reference)						
	Male	.41	1.12	.80–1.56	.02	.98	.70–1.37
*Cohabitation status*	Cohabiting (reference)						
	Alone	.02	1.03	.61–1.75	.05	1.06	.63–1.80
*Perceived greater risk*	Yes (reference)						
	No	.64	.81	.48–1.37	.17	.90	.53–1.52
	Unsure	.37	.88	.58–1.34	.10	.93	.61–1.42
*Employment status*	Furloughed (Reference)						
	Employed full time	3.20	.56	.30–1.06	1.61	.66	.36–1.29
	Employed part time	2.57	56	.27–1.14	.61	.76	.37–1.53
	Unemployed looking for work	.05	1.14	.36–3.59	.73	1.65	.53–5.23
	Unemployed not looking for work	.11	.82	.25–2.66	.12	.81	.25–2.64
	Retired	1.06	.54	.17–1.74	1.31	.51	.16–1.62
	Student	3.10	.58	.32–1.06	1.30	.71	.39–1.29
	Disabled	1.02	2.63	.40–17.15	.20	1.53	.24–9.99
*Socialisation satisfaction*		13.11	1.30**	1.10–1.50	11.50 **	1.28	1.10–1.48
*Depression*		3.17	.96	.92–1.00	2.54	.97	.92–1.01
*Anxiety*		2.84	1.05	.99–1.12	1.12	1.03	.97–1.10
*Stress*		5.06	.94*	.90 –.99	3.52	.95	.91–1.00
*Task-load*		5.87	1.05*	1.01–1.10	3.83	1.04	1.00–1.09
*Physical activity*		.12	1.02	.90–1.16	.01	.99	.87–1.13
*Change of routine*		.65	1.08	.90–1.30	.76	1.09	.90–1.31

## Discussion

This study examined the passage of time during the UKs lockdown period in response to the Covid-19 pandemic. Specifically, the study aimed to establish factors which contributed to the relative speeding up and slowing down of the passage of time during this period.

The results show that, for many people, the passage of time felt distorted during the lock-down period in comparison with normal. For both the day and the week POTJs, participants were more likely to respond that the speed of the passage of time was distorted in comparison with normal, than to respond that time was passing at a normal rate. Interestingly, time did not distort in a single direction for all participants (i.e. faster or slower), instead, experiences of time passing more quickly or more slowly than normal were split almost evenly across those who experienced a distortion of the passage of time. Social and physical distancing measures therefore appear to have different effects on the passage of time in different people. Despite this, the experience of distortion to the passage of time appears to be consistent within individuals, as demonstrated by the strong positive correlation between POTJ-day and POTJ-week responses. People who experienced fast days were therefore also likely to experience fast weeks, whereas people who experienced slow days were more likely to also experience slow weeks.

The speed of the passage of time during the lockdown was related to a number of factors. How quickly time was felt to pass during a day was related to levels of depression and stress, satisfaction with current levels of social interaction and participant age. However, regression analysis revealed that age, satisfaction with levels of social interaction, stress and task load were the only factors which were predictive of the speed of the passage of time. The likelihood of the day passing more slowly increased with increasing stress, reduced task load, increasing age and decreasing satisfaction with current levels of socialisation. The speed of the passage of time for the week correlated with depression, stress, age and satisfaction with socialisation. Subsequent regression analysis however revealed that only age and satisfaction with socialisation levels were predictive of the speed at which time passed during the week. Again, increasing age and greater dissatisfaction with levels of social interaction increased the likelihood of time passing more slowly across the week. Occupation, cohabitation status, perceived risk to Covid-19, anxiety, level of physical activity and extent to which daily life has changed due to the lock-down were unrelated to both passage of time judgments. Therefore, although the experience of the passage of time seemed broadly consistent across the two epochs (day and week), different factors predicted the speed of the passage of time for these periods.

These findings enable us to draw a number of conclusions about the passage time during the UK Covid-19 lockdown. Firstly, during times of social and physical distancing, satisfaction with current levels of social interaction is a significant predictor of the speed of the passage of time. Greater dissatisfaction with levels of social interaction are associated with a slowing of the passage of time whereas greater satisfaction with the level of social interaction are associated with an increase in the speed of the passage of time. Previous research has suggested that affective factors have a consistent effect on the passage of time, with positive affect being associated with a faster passage of time and negative affect being associated with a slowing of the passage of time [1, 14, 15, 19]. The findings of this study support these suggestions; greater stress was associated with a slower passage of time as was greater dissatisfaction with social interaction, which is a form of negative affect, which in this and other studies [47] is highly correlated with levels of depression and anxiety. Therefore, in times of societal upheaval, emotion influences the passage of time in broadly comparable ways to during normal times. The absence of a predictive effect of depression or anxiety on the passage of time in the current study does, however, suggest that more situation-specific affective measures may be better predictors of temporal experience than more general ones. Indeed, a key question for future research is whether social satisfaction is predictive of the passage of time during normal daily life. If it is not, this perhaps suggests that how we experience time is not shaped by global affective factors but, instead, that the passage of time is determined by event specific factors centring on the most salient changes during different periods.

Secondly, there are some differences in the factors which affect the subjective speed of the passage of time over shorter (day) and longer (week) epochs. Although age and satisfaction with levels of social interaction predicted both day and week POTJs, stress and task load only predicted day POTJs. For POTJ-day, increasing stress was associated with a slowing of the passage of time whereas increasing task load was associated with a faster passage. The observed association between increased task load and a faster passage of time supports observations from laboratory studies exploring the effect of information processing load on passage of time judgements [1, 10, 11, 15]. Task-load therefore has comparable effects in the real-world and laboratory studies. Increased task load is thought to hasten the passage of time because it reduces boredom and the amount of attention paid to time [10, 11]. Increasing the task demands of the day therefore appears to be one way in which people can hasten the passage of time during the lockdown. However, because increased stress is associated with a slower passage of time, and increased task load can be associated with increased stress, it is possible that increasing task demands to a point that stress is induced may actually result in a slowing of the passage of time. Future research should examine the potential bidirectional effects of task-load and stress on time experience.

It is unclear why stress and task-load only affected the day and not week POTJ. One possibility is that participants struggled to accurately remember their average task load, as a consequence, responded based primarily on their current day experience. Alternatively, large variations in levels of stress and task-load across the week could have made it difficult for participants to produce a meaningful average. Thus, inaccuracies in the reporting of the task load may have contributed to the absence of an effect of this variable for POTJ-week.

Finally, the findings of this study suggest that during times of social and physical distancing, age is a significant predictor of the passage of time, with increasing age being associated with an increasingly slow passage of time. Specifically, whilst people aged 18 to 60 appear to experience time in a comparable way, people over the age of 60 appear to experience a relative slowing of the passage of time during lockdown when compared to those under the age of 60. This contrasts with recent studies conducted under “normal” circumstances which typically show that the passage of time does not differ between younger and older adults [14, 15]. However, it is important to note there are a number of key methodological differences between this study and Droit-Volet and colleagues, with Droit-Volet’s work primarily measuring the passage of time in the present moment, rather than the retrospective passage of time across days and weeks as in this study. The differing findings may therefore reflect differing effects of age on different epochs of passage of time judgements. However, it is also possible that the differing findings reflect the effects of social and physical distancing on the passage of time. There is some evidence that older adults can experience a slowing of the passage of time, but only when they are living in residential care homes [21]. This slowing of the passage of time for residential care home residents is thought to be due to being in the residential home itself and the definitive change that brings to life, rather than age related change in cognition or affect. Together, the current study and Droit-Volet (2019) [21] perhaps suggest that age effects on the passage of time become most apparent when there is a significant change in normal life. Critically, together, they also suggest that rather than speeding up, time may actually pass more slowly in elderly group.

### Limitations

The current study demonstrates the influence of affective experience, task load and demographic variables on the experience of time during the Covid-19 lockdown. Although these variables were selected on the basis of previous research, they only accounted for a relatively small proportion of the variance in POTJs. This suggests that there are many additional variables, not included in this study, which influenced the passage of time. Laboratory studies show that impulsivity and sense of entitlement affect the subjective passage of time [28, 48]. In particular, greater impulsivity and a greater sense of entitlement have been associated with the slowing of the passage of time, particularly during boredom. It is therefore possible that these factors may also influenced the passage of time during lockdown.

Another factor known to affect the passage of time during normal daily life is alcohol consumption [1]. Alcohol consumption is believed to be increasing during the current social and physical distancing measures [49]. This raises the possibility that atypical alcohol consumption rates during lockdown may contribute to the experience of the passage of time during this period. Future research should therefore explore the effect of drug and alcohol use on the passage of time during lockdown.

Finally, the current study was limited to people currently living in the UK and did not therefore examine cross-cultural experiences of time during lock-down. To date, there is little research examining cross-cultural differences in passage of time judgements during normal daily life and atypical daily life. Language and culture are however known to influence the way in which time is described and experienced [50] and it is therefore possible that social and physical distancing measures may have different effects on the passage of time in different cultures.

## Conclusion

The results of this study suggest that the UK’s Covid-19 social and physical distancing measures had a significant effect on peoples experience of the passage of time in comparison to normal times (pre-lockdown). This suggests that fundamental changes in day-to-day life distort our experience of time. Age and level of satisfaction with social interaction were predictive of the relative speed that time passed during the lockdown in comparison to normal. Experiencing time as passing quickly during the lockdown was therefore associated with reduced age and increased satisfaction with current levels of social interaction. Stress and task load also affected the passage of time day judgment with increased task load and reduced stress being associated with a faster passage of time. Whilst these findings are reminiscent of those observed in studies of the passage of time during normal daily life, the predictive value of social satisfaction, as apposed to anxiety and depression, highlights the benefit of measuring situation specific variables when understanding the passage of time. Furthermore, the age effects observed reopen the debate about whether the passage of time really does distort with increasing age.

## Supporting information

S1 File The questionnaire. (DOCX)

S1 Data Supporting data. (SAV)
