# Supplementary material for: The passage of time during the UK Covid-19 lockdown
Source: PLoS One. 2020 Jul 6;15(7):e0235871. doi: 10.1371/journal.pone.0235871 (PMC7337311; doi:10.1371/journal.pone.0235871)
Supplement: S1 File — (DOCX) [file pone.0235871.s001.docx]

Questionnaire: The passage of time during the UK Covid-19 lockdown

1. How old are you?______________
2. Please state your gender.______________________
3. Which best describes your current employment status?
4. Employed full time
5. Employed part time
6. Unemployed looking for work
7. Unemployed not looking for work
8. Retired
9. Student
10. Disabled
11. Furloughed
12. How many people live in your household excluding yourself?_________
13. Since the covid-19 lockdown, how satisfied are you with your daily level of social interaction?
    1. Extremely satisfied
    2. Somewhat satisfied
    3. Neither satisfied nor dissatisfied
    4. Somewhat dissatisfied
    5. Extremely dissatisfied
14. Since the covid-19 lockdown, how would you describe your level of physical activity?
    1. Extremely active
    2. Somewhat active
    3. Quite active
    4. Rarely active
    5. Inactive
15. To what extent do you agree with the following statement: "My daily routine has changed a lot as a result of the covid-19 lockdown?
    1. Strongly agree
    2. Somewhat agree
    3. Neither agree nor disagree
    4. Somewhat disagree
    5. Strongly disagree
16. Do you consider yourself to be at high risk from covid-19?
    1. Yes
    2. No
    3. Unsure
17. Thinking about today, how quickly has time felt like it is passing in comparison with normal (i.e. before lockdown)?
    1. Extremely slow
    2. Somewhat slower
    3. A little slower
    4. As normal
    5. A little faster
    6. Somewhat faster
    7. Extremely fast.
18. Thinking about this week, how quickly has time felt like it was passing in comparison to normal (i.e. before lockdown)?
    1. Extremely slow
    2. Somewhat slower
    3. A little slower
    4. As normal
    5. A little faster
    6. Somewhat faster
    7. Extremely fast.
19. Which country are you currently in?____________________________

Participants then completed the following two questionnaires:

DASS-21 (Lovibond PF, Lovibond SH. The structure of negative emotional states: Comparison of the Depression Anxiety Stress Scales (DASS) with the Beck Depression and Anxiety Inventories. Behaviour research and therapy. 1995 Mar 1;33(3):335-43).

NASA – TLX Hart SG, Staveland LE. Development of NASA-TLX (Task Load Index): Results of empirical and theoretical research. In Advances in psychology 1988 Jan 1 (Vol. 52, pp. 139-183). North-Holland.
